# Access to specialist ataxia centres in Europe: cross-sectional surveys in the United Kingdom, Italy, and Germany

**DOI:** 10.3389/frhs.2026.1787866

**Published:** 2026-06-24

**Authors:** Suzanne Booth, Julie Vallortigara, Catherine Hurt, Julie Greenfield, Barry Hunt, Paola Giunti

**Affiliations:** 1 Ataxia Centre, Queen Square Institute of Neurology, Department of Molecular and Movement Neurosciences, University College London, United Kingdom; 2 City St George’s, University of London, United Kingdom; 3 Ataxia UK, London, United Kingdom

**Keywords:** care pathway, patient survey, progressive ataxias, rare diseases, specialist centre

## Abstract

**Background:**

Progressive ataxias are a diverse and rare group of neurodegenerative conditions characterised by unsteadiness, incoordination and slurred speech. Additional neurological features and pathology outside the central nervous system may also be present.^1^ Optimal management of these complex conditions requires access to specialist diagnostics and treatment via Specialist Ataxia Centres (SACs). Greater understanding of factors impacting on SAC access may help to maximise utilisation.^2^

**Aim:**

This study explored factors associated with referral to SACs or discontinued access.

**Methods:**

Responses to cross-sectional surveys conducted separately in three European countries were collated. The national surveys were conducted at different times and the question format evolved, requiring responses to be harmonised. The 550 individual responses were examined for associations between SAC access and respondent characteristics.

**Results:**

Respondents with a genetic diagnosis were observed to have greater representation in SACs in Italy and Germany than in the UK, with this contrast being most prominent with a diagnosis of Friedreich's ataxia. Respondents with Idiopathic Cerebellar Ataxia also had significantly lower proportion of referrals in the UK relative to Germany and Italy. Fewer reported comorbidities, being in employment, and shorter travel time to SACs were associated with reported SAC attendance or referral. Increasing mobility impairment was associated with reported discontinuation of SAC attendance. No statistically significant associations were observed between SAC attendance and referral source, time since diagnosis, or reported impact of ataxia on daily living.

**Conclusions:**

Statistically significant differences between SAC and non-SAC respondent cohorts were observed, as well as variations in SAC access between countries. These findings highlight factors that may be relevant to SAC utilisation. The results suggest potential areas for further investigation, including strategies to support access, such as telehealth, and may inform the design of future multinational studies.

## Introduction

The progressive ataxias are a clinically and aetiologically diverse group of rare neurodegenerative conditions resulting in significant burden of disease (1, 2). Ataxia may arise from sensory or cerebellar impairment and may be inherited or acquired. To date, more than 100 distinct subtypes of inherited ataxia have been identified which are classified according to their genetic loci (1).

Ataxia is characterised by reduced coordination, gait changes and difficulties with speech and swallowing. Further neurological features (e.g., spasticity) or disease outside the central nervous system, such as the cardiomyopathy seen in Friedreich's ataxia (FA), may also manifest. Mobility and communication skills are significantly reduced, compromising quality of life and potentially resulting in premature death (2, 3). There is substantial variation in age of onset, with the spinocerebellar ataxias (SCA) characteristically presenting in adult life, however conditions such as Friedreich's ataxia (FA) and Episodic ataxia (EA) can be juvenile-onset (<20 years), early adult-onset (20–40 years), or late adult-onset (>40 years) (4, 5).

The estimated global prevalence rate of progressive ataxia in children is 26 cases per 100,000 (6). For Inherited cerebellar ataxia (Inherited CA) a prevalence rate of 2.7 to 3.3 cases per 100,000 for dominant and recessive hereditary cerebellar ataxia is observed (6). In the United Kingdom it is estimated that more than 10,000 people are living with a progressive ataxia, a figure expected to increase with an ageing population (7, 8). Variable and overlapping symptoms and signs pose diagnostic challenges. Idiopathic cerebellar ataxia (Idiopathic CA), where an underlying cause has not been identified, is reported to have an estimated prevalence rate of 8.4 cases per 100,000 (9).

Current disease-modifying agents have limited application and benefit (10). Timely diagnostics, and access to highly customised symptomatic management and therapy interventions are therefore crucial to maximise independence and quality of life. Traditionally, neurologists have held a pessimistic view to managing progressive ataxias, as with other neurodegenerative diseases lacking curative treatments. There is however growing evidence that coordinated, specialist multidisciplinary input improves diagnostic success and long-term management of these conditions, thereby decreasing disease burden (11, 12).

Significant barriers exist to accessing appropriate diagnostic and treatment options, due to the lack of awareness and understanding of these rare conditions within primary and secondary care settings, although effective management expertise is available via Specialist Ataxia Centres (SACs) in the United Kingdom and Europe (13, 14). SACs are centres of excellence, employing the expertise of specialist neurologists and nurses, geneticists, physiotherapists, occupational therapists, speech and language therapists, along with networks to relevant medical disciplines, to provide a coordinated approach to diagnostics, symptom management and research (15).

Optimal care is not being uniformly received by people with ataxia, as many are not accessing the resources available via SACs (13, 14). Previous work by Vallortigara et al. (2023) (16), has described patient pathways for rare diseases across Europe, highlighting variability in access to ataxia specialist services. A better understanding of the factors that impact on SAC access is crucial to improving utilisation of specialised care.

While there is a paucity of evidence regarding referral practices to SACs, in other neurological conditions referral variation is observed to occur due to patient population differences in different settings. In Multiple Sclerosis (MS), it has been identified that those with greater disease severity are more likely to receive care in specialised settings, whereas less complex cases are often cared for in general outpatient or private settings (17). Further variation may stem from physician or patient preferences and opinions (18, 19) (e.g., treatment expectations), or barriers to care (e.g., due to discontinued referrals) (20).

It is proposed that the most detrimental factors leading to variations in SAC access may concern socio-economic or treatment pathway challenges, as they may lead to underutilisation of optimal care, and adversely impact on future health outcomes (17). Several studies report similar barriers in other neurological conditions. Almost 1 in 4 people with MS do not receive MS specialist support, with vulnerable populations such as ethnic minorities, those with longer travel time to specialist services, or greater disability being less likely to see a neurologist (21).

The study we present in this manuscript is part of a bigger project, using anonymous surveys distributed in three countries with well-established SACs in operation (the United Kingdom, Italy and Germany). The surveys were designed to gather information on patient care pathways and the experiences of SAC access (16, 22, 23). The present study analysed survey data with the objective of:

1. Defining key characteristics of survey respondents in the United Kingdom, Italy and Germany with regard to ataxia diagnosis, disease burden, comorbidity and a selection of socio-demographic variables.
2. Exploring whether significant differences exist between SAC and non-SAC respondent cohorts, whether national variations can be observed, and identifying possible predictors for referral to SAC or discontinued SAC attendance.

## Methods

### Study population

At the time of survey distribution in the UK there were two SACs (London and Sheffield); nine in Germany (Lunbeck, Munich, Tubingen, Bonn, Essen, Aachen, Berlin, Dusseldorf and Magdeburg); and eleven in Italy [Florence, Milan, Messina, Naples, Rome (two centres), Siena, Turin, Pisa, Genova and Bologna].

People with ataxia, or carers responding on their behalf, aged 16 years or over, were eligible to participate. In each country patients’ groups (Ataxia UK, German patient groups DHAG and Friedreich Ataxie Förderverein e.V., and Italian patient group AISA) assisted with recruitment of participants. The survey was mainly disseminated in the UK via online via patient group platforms (mailing list, website, magazine and social media). In Germany and Italy surveys were initially distributed via patients’ groups, however as this did not result in a strong initial uptake, participants were additionally recruited by clinicians directly via SACs.

The open-access online tool Surveymonkey® (http://www.surveymonkey.com) was used to administer the survey. Invitation emails sent to potential participants provided a patient information sheet and a weblink directly to SurveyMonkey. Patients without online access were invited to complete the survey in paper form, and data was entered on SurveyMonkey.

The survey received ethical approval via the Integrated Research Application System (Reference 19/EE/0030) prior to survey distribution. The proposed study was registered with City University's ‘Research Manager’ and received approval from the University of London's School of Health & Psychological Sciences research ethics committee (Reference ETH2223-0572). Ethical approval for an anonymised survey was not required In Germany and Italy.

### Study design

This exploratory study analysed data obtained via a cross-sectional survey of people with ataxia in the UK between March to May 2019, in Germany from February to October 2020, and in Italy from May to September 2021. Widespread disruption during the COVID-19 pandemic meant that data collection occurred at different time points across countries and over variable durations.

### Survey

Patient group representatives, specialist ataxia clinicians, health economists and representatives of pharmaceutical companies were involved in the design of the survey. The final version of the survey questionnaire had 64 questions relating to the following topics: demographics, diagnosis, referral, patients’ encounters with healthcare professionals, treatment received and patient satisfaction. The questionnaire mainly consisted of close-ended questions with a limited set of predefined response options. Survey questions were chosen based on the experience of the research study team as described previously and a review of the literature. The age brackets were chosen reflecting different stages of life: young adult, employment age, retirement, and old age.

Study context and medical terms used were outlined at the beginning of the survey which was circulated first in the UK (Appendix 1). Subsequently local language versions of the survey were translated by bilingual clinicians and patient group representatives for dissemination in Italy and Germany (Appendices 2 and 3). These incorporated several additional questions, as well as some minor revisions to the structure or wording of a number of questions, including amendments to the wording of some multiple-choice options. Amendments were based on data collected via the UK survey and the input of clinicians and patient representatives in both Germany and Italy.

### Outcome measures/comparator groups

As Inherited ataxias can be viewed as a distinct group, for analysis purposes Inherited CA, FA and EA were amalgamated into a single group defined as ‘Genetic Diagnoses’. Those respondents who had not received a genetic diagnosis or had yet to be tested were defined as ‘All Other Diagnoses’.

Not all respondents who indicated they had been referred to an SAC had actually attended an SAC at the point of completing the survey. It was therefore necessary to separate referral status from attendance status.

Participants were grouped according to self-reported current access of specialist ataxia centres (Current SAC), those who have discontinued access to an SAC (Discontinued SAC), those potentially referred/unsure of referral status but yet to attend (Pending SAC) and those who have not attended an SAC (Never SAC).

An aggregation exercise was performed to simplify the history of respondent SAC attendance, combining ‘Current SAC’ and ‘Discontinued SAC’ into a “Has Attended SAC” category. Respondents who have experienced the mixed pathway of discontinued SAC attendance may not have discontinued out of choice (e.g., closure of an SAC in the UK), and their period of SAC attendance may have impacted disease outcomes. The survey did not address how long respondents attended an SAC before discontinuing. This aggregated category therefore permits clearer observation of any impact SAC attendance may have had regardless of discontinuation, with ‘Pending SAC’ and ‘Never SAC’ being mapped to “Not Attended SAC”.

A similar amalgamation was performed according to referral status to create a ‘Referred’ group, combining ‘Current SAC’, ‘Previous SAC’, and ‘Pending SAC’ cohorts and those that have not been referred allocated to ‘Not Referred’.

### Data preparation

Responses were removed where respondents did not provide positive responses to the three mandatory screening questions (questions 3, 4 and 5; see Appendix 2). The remaining survey questions were not obligatory, and incomplete surveys were therefore not removed from the analysis.

Multiple choice questions that included a free text response option were reviewed individually to ensure the overall question was answered. Where an inconsistent, but clear, response was observed in a free text response the respondent's prior response was amended to the correct category to safeguard the value of the survey. A large number of minor discrepancies related to language translation in the multiple-choice options required manual reformatting to obtain consistent results across the three surveys. Further variations in available response options between the UK, Italian and German surveys required harmonisation prior to conducting the statistical analysis. Inconsistencies between survey response options for travel time to an SAC required harmonisation to create four consistent categories: <1 h, 1–2 h, 2–4 h and 4 + hours.

The surveys were distributed on different dates, and were in circulation for different periods of time. Italian and German respondents were asked to provide a date of diagnosis, whereas UK respondents were asked to pick a band of years elapsed. To harmonise responses across all three surveys it was necessary to convert Italian and German responses into the UK format. This required choosing a single proxy date to represent the Italian and German survey date so that a time period could be calculated per respondent.

Respondents were requested to rate their mobility on a score of 1–9, based on a validated ataxia rating scale (24). This resulted in a large number of degrees of freedom when running statistical analyses, with some cells containing no respondents, or very few. The nine mobility responses were therefore mapped across to three broad categories: Walking unassisted (1–3); Walking with assistance (4–6), and Unable to Walk (7–9).

### Statistical analysis

A subset of questions from the surveys were used to analyse data on the following parameters: (1) Clinical and socio-demographic details; (2) Disease burden, stage of disease, comorbidities; (3) Specialist ataxia centre (SAC) status. Microsoft Excel was used to perform data cleaning and response harmonisation, as well as to map multiple responses across to grouped categories. Statistical analyses were then conducted using IBM SPSS (Statistical Package for Social Sciences) version 25.

Results were prepared as tabulated descriptive statistics and presented as numbers (N) and percentage (%) of total respondents per question. Pearson's chi-squared test (*χ*^2^) was used to identify statistically significant associations within the categorical data. For all the analyses, *p*-values <0.05 were considered to be statistically significant. In order to obtain a valid Pearson's chi-squared test (*χ*^2^) it was sometimes necessary to reduce the degrees of freedom by mapping responses to a smaller number of categories:

- For ataxia diagnosis analysis, “Other unknown diagnosis” was amalgamated with the very small number in “Other known cause” to create a single variable “Ataxia other types” as there was no analytical benefit to keeping these very widely defined groups separate.
- Similarly, for non-ataxia related comorbidities the responses were grouped according to number of conditions reported: 0,1,2 + and Unsure. The Italian and German surveys included psychiatric disorders as an additional prompted option that was not available in the UK survey. To harmonise responses across all three surveys it was necessary to remove this option for the analysis.

Some survey questions only garnered a handful of responses for options such as “Unsure”, “No Response” and “Other”. On occasion it was necessary to exclude these respondents from the sample to ensure that cells with an expected frequency of less than 5 did not compromise the results when using Pearson's chi-squared test (*χ*^2^). Fisher's Exact Test was not a feasible alternative in most cases on account of the large number of answer options available to respondents.

## Results

### Survey respondent characteristics

As shown in Table 1, this study included 550 respondents across the three countries: UK = 277; Italy = 174; Germany 99. The majority of respondents in the UK were aged over 60 years (56.3%), whereas the cohorts in Germany and Italy were younger, with the 30–59 years category being most populous (59.6% and 66.5% respectively). In the 80 + category, 12 of the 13 respondents were UK respondents (Supplementary Figure 1). No statistically significant differences in gender distribution were observed.

**Table 1:** Survey respondent demographics (*N* = 550).

	Germany		Italy		UK		Total	
	N	(%)	N	(%)	N	(%)	N	(%)
**Survey Respondents (*N*** **=** **550)**	99	(18.0%)	174	(31.6%)	277	(50.4%)	**550**	**(** **100.0%)**
Gender								
Female	47	(47.5%)	93	(53.8%)	142	(52.6%)	**282**	**(** **52.0%)**
Male	52	(52.5%)	80	(46.2%)	128	(47.4%)	**260**	**(** **48.0%)**
*χ*^2^ (2, *N* = 542) = 1.06, *p* *=* .587	***99***	***(100.0%)***	***173***	***(100.0%)***	***270***	***(100.0%)***	***542***	***(100.0%)***
Age Distribution								
16–29	12	(12.1%)	22	(12.7%)	12	(4.4%)	**46**	**(** **8.5%)**
30–59	59	(59.6%)	115	(66.5%)	106	(39.3%)	**280**	**(** **51.7%)**
60–80	28	(28.3%)	35	(20.2%)	140	(51.9%)	**203**	**(** **37.5%)**
80+	0	(0.0%)	1	(0.6%)	12	(4.4%)	**13**	**(** **2.4%)**
***χ*****^2^ (6, *N*** **=** **542)** **=** **1.06, *p*** ***=*** **<.001**	***99***	***(100.0%)***	***173***	***(100.0%)***	***270***	***(100.0%)***	***542***	***(100.0%)***
Diagnosis								
Inherited CA	53	(56.4%)	58	(36.3%)	102	(37.2%)	**213**	**(** **40.3%)**
Idiopathic CA	6	(6.4%)	29	(18.1%)	117	(42.7%)	**152**	**(** **28.8%)**
Friedreich's ataxia (FA)	14	(14.9%)	56	(35.0%)	27	(9.9%)	**97**	**(** **18.4%)**
Ataxia (other types)	19	(20.2%)	15	(9.4%)	22	(8.0%)	**56**	**(** **10.6%)**
Episodic ataxia (EA)	2	(2.1%)	2	(1.3%)	6	(2.2%)	**10**	**(** **1.9%)**
***χ*****^2^ (8, *N*** **=** **528)** **=** **94.60, *p*** ***=*** **<.001**	***94***	***(100.0%)***	***160***	***(100.0%)***	***274***	***(100.0%)***	***528***	***(100.0%)***
SAC Status								
Current SAC	48	(56.5%)	83	(58.9%)	72	(27.2%)	**203**	**(** **41.3%)**
Discontinued SAC	13	(15.3%)	30	(21.3%)	48	(18.1%)	**91**	**(** **18.5%)**
Pending SAC (including Unclear)	3	(3.5%)	1	(0.7%)	17	(6.4%)	**21**	**(** **4.3%)**
Never SAC	21	(24.7%)	27	(19.1%)	128	(48.3%)	**176**	**(** **35.8%)**
***χ*****^2^ (6, *N*** **=** **491)** **=** **61.65, *p*** ***=*** **<.001**	***85***	***(100.0%)***	***141***	***(100.0%)***	***265***	***(100.0%)***	***491***	***(100.0%)***
SAC Referral								
Referred	61	(71.8%)	114	(80.9%)	137	(51.7%)	**312**	**(** **63.5%)**
Not Referred	21	(24.7%)	27	(19.1%)	128	(48.3%)	**176**	**(** **35.8%)**
Referral Unclear	3	(3.5%)	0	(0.0%)	0	(0.0%)	**3**	**(** **0.6%)**
***χ*****^2^ (4, *N*** **=** **491)** **=** **53.11, *p*** ***=*** **<.001**	***85***	***(100.0%)***	***141***	***(100.0%)***	***265***	***(100.0%)***	***491***	***(100.0%)***
Time Elapsed Since Diagnosis								
<0.5yr	1	(1.2%)	3	(2.2%)	6	(2.2%)	**10**	**(** **2.0%)**
0.5yr-<1yr	2	(2.4%)	1	(0.7%)	11	(4.1%)	**14**	**(** **2.9%)**
1yr-<2yr	7	(8.2%)	7	(5.2%)	22	(8.1%)	**36**	**(** **7.3%)**
2yr-<5yr	16	(18.8%)	20	(14.8%)	63	(23.2%)	**99**	**(** **20.2%)**
>=5yr	59	(69.4%)	104	(77.0%)	169	(62.4%)	**332**	**(** **67.6%)**
*χ*^2^ (8, *N* = 491) = 11.30, *p* *=* .185	***85***	***(100.0%)***	***135***	***(100.0%)***	***271***	***(100.0%)***	***491***	***(100.0%)***
Count of Comorbidities								
None	33	(37.9%)	111	(69.8%)	122	(52.6%)	**266**	**(** **55.6%)**
1	37	(42.5%)	37	(23.3%)	70	(30.2%)	**144**	**(** **30.1%)**
2+	17	(19.5%)	11	(6.9%)	40	(17.2%)	**68**	**(** **14.2%)**
***χ*****^2^ (4, *N*** **=** **478)** **=** **27.13, *p*** ***=*** **<.001**	***87***	***(100.0%)***	***159***	***(100.0%)***	***232***	***(100.0%)***	***478***	***(100.0%)***
Employment (*N* = 518)								
In Education	4	(4.4%)	14	(8.9%)	3	(1.1%)	**21**	**(** **4.1%)**
In Work	29	(31.9%)	51	(32.3%)	42	(15.6%)	**122**	**(** **23.6%)**
Retired	15	(16.5%)	22	(13.9%)	78	(29.0%)	**115**	**(** **22.2%)**
Left/Unable to Work (ataxia related)	33	(36.3%)	52	(32.9%)	115	(42.8%)	**200**	**(** **38.6%)**
Other	10	(11.0%)	19	(12.0%)	31	(11.5%)	**60**	**(** **11.6%)**
***χ*****^2^ (8, *N*** **=** **518)** **=** **44.26, *p*** ***=*** **<.001**	***91***	***(100.0%)***	***158***	***(100.0%)***	***269***	***(100.0%)***	***518***	***(100.0%)***
Mobility (question not in UK survey)								
Walking unassisted	24	(24.5%)	13	(7.5%)			**37**	**(** **13.6%)**
Walking with assistance	49	(50.0%)	88	(50.6%)			**137**	**(** **50.4%)**
Unable to walk	25	(25.5%)	73	(42.0%)			**98**	**(** **36.0%)**
***χ*****^2^ (2, *N*** **=** **272)** **=** **18.06, *p*** ***=*** **<.001**	***98***	***(100.0%)***	***174***	***(100.0%)***			***272***	***(100.0%)***
Impact on Daily Living (question not in UK survey)								
Does not affect me	4	(4.2%)	4	(2.5%)			**8**	**(** **3.1%)**
Causes occasional problems	16	(16.8%)	18	(11.0%)			**34**	**(** **13.2%)**
Causes frequent problems restricting my activities	25	(26.3%)	55	(33.7%)			**80**	**(** **31.0%)**
Causes constant problems that restricted me most or all of the time	50	(52.6%)	86	(52.8%)			**136**	**(** **52.7%)**
*χ*^2^ (3, *N* = 258) = 3.20, *p* *=* .362	***95***	***(100.0%)***	***163***	***(100.0%)***			***258***	***(100.0%)***

**Figure 1:**
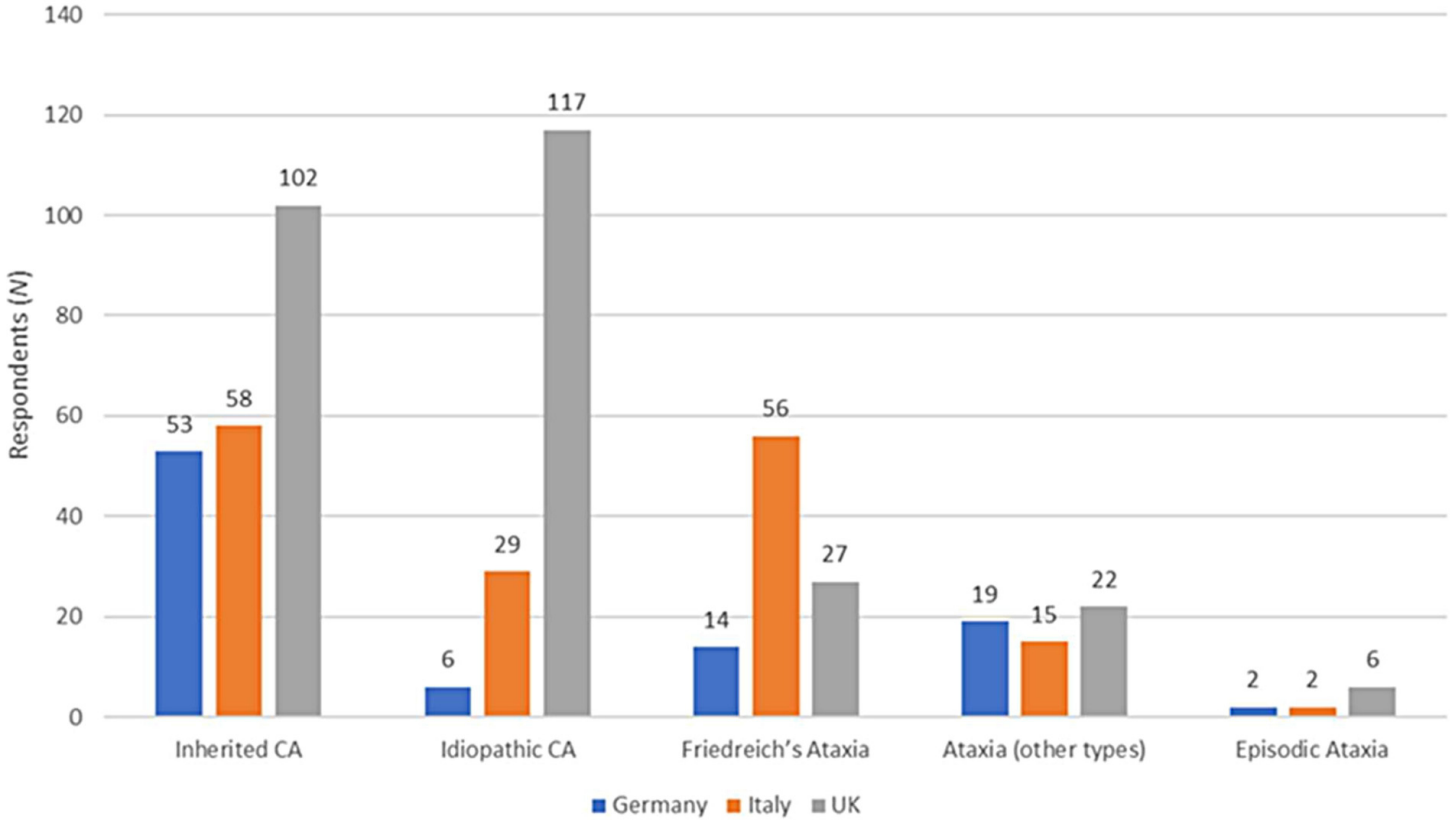
Ataxia diagnosis of survey respondents (*N* = 528) Higher proportion of idiopathic CA was observed in the UK respondents whereas in Italy a higher proportion of FA was observed [Pearson chi-squared test *χ*^2^ (8, *N* = 528) = 94.60, *p* = <.001].

Regarding type of ataxia, 40.3% of the total cohort reported a diagnosis of Inherited CA. Between nations, statistically significant variations (at the 0.05 level, relative to the Inherited CA proportion) in ataxia type were observed, with a much higher proportion of Idiopathic CA in the UK sample (42.7% vs. 28.8% overall), and the Italian cohort displaying a high proportion of FA (35.0% vs. 18.4% overall) (Figure 1). A significant proportion of respondents were diagnosed more than 5 years ago (67.6%). Of the total cohort, 35.8% had never been referred to an SAC.

The majority of respondents were not in employment or education, with a significant percentage having left work due to their diagnosis (38.6%) or reporting retirement (22.2%). Due to the broad age categories used in the surveys it was not feasible to compare the distribution of respondents’ ages with the age distribution of all citizens in each country (Table 1).

The Italian and German surveys included an additional question on current level of disability including impact on activities of daily living (ADL) and mobility. The majority (83.7%) reported ataxia causing frequent or constant problems restricting ADL. Similarly, significant mobility restrictions were reported for the majority of both countries’ respondents, with 50.4% requiring assistance to mobilise and 36% unable to walk (Table 1).

### SAC cohort characteristics

Separate questions in the surveys addressed whether respondents were attending an SAC and whether they had been referred to an SAC. There was a high degree of overlap between the response distributions, but they were not identical. Chi-squared tests identified a statistically significant association (<.01) between SAC status and individual country referral rates, with a much higher proportion of respondents in Germany and Italy reporting to be Current SAC or Referred to SAC than in the UK (Supplementary Figure 2). Across the three countries, 203 ataxia participants responded that they were currently attending an SAC (“Current SAC”). The proportion of participants currently attending an SAC in the UK was much lower than observed in the two other countries, with 27.2% of respondents reported to be currently attending an SAC. Germany and Italy by comparison reported more people with ataxia currently receiving care via an SAC (56.5% and 58.9% respectively) (Table 1).

A further 91 respondents indicated that they had previously attended an SAC but were no longer attending. These respondents were combined with “Current SAC” to create the “Has Attended SAC” cohort (*N* = 294) of people who have experienced the mixed pathway. This enabled observation of any potential impact that SAC attendance may have, whether discontinued or current. Within that cohort, there were 61 respondents from Germany (representing 71.8% of the German group), 134 from Italy (80.1% of the Italian group), and 120 from the UK (45.3% of the UK group).

### Age distribution

Significant association was observed between referral status and age [*χ*2 (3, *N* = 486) = 13.19, *p* = .004]. Respondents in the 30–59 age group had a statistically significant higher weighting, with a lower referral rate seen in the 60–80 group.

### Impact of ataxia on daily living

Neither SAC referral nor SAC attendance displayed any statistically significant association with the reported impact of ataxia on daily living. Respondents who reported their condition “Does not affect me” were as likely to be ‘Current SAC’ or ‘Referred’ to SAC as those experiencing “Constant problems”.

### Variations by SAC Status

Differences in survey samples according to SAC status, including ‘Current SAC’, ‘Discontinued SAC’, ‘Pending SAC’ and ‘Never SAC’ were analysed. The same analysis was performed again, substituting SAC attendance status with referral status, in order to incorporate up to 21 respondents who may have been referred but not yet attended an SAC. Grouping the SAC Status into a smaller number of SAC Referral categories changed the chi-squared result from *χ*2 (6, *N* = 491) = 61.65, *p* = <.001 to *χ*2 (4, *N* = 491) = 53.11, *p* = <.001.

### Count of non-ataxia related comorbidities

**Figure 2:**
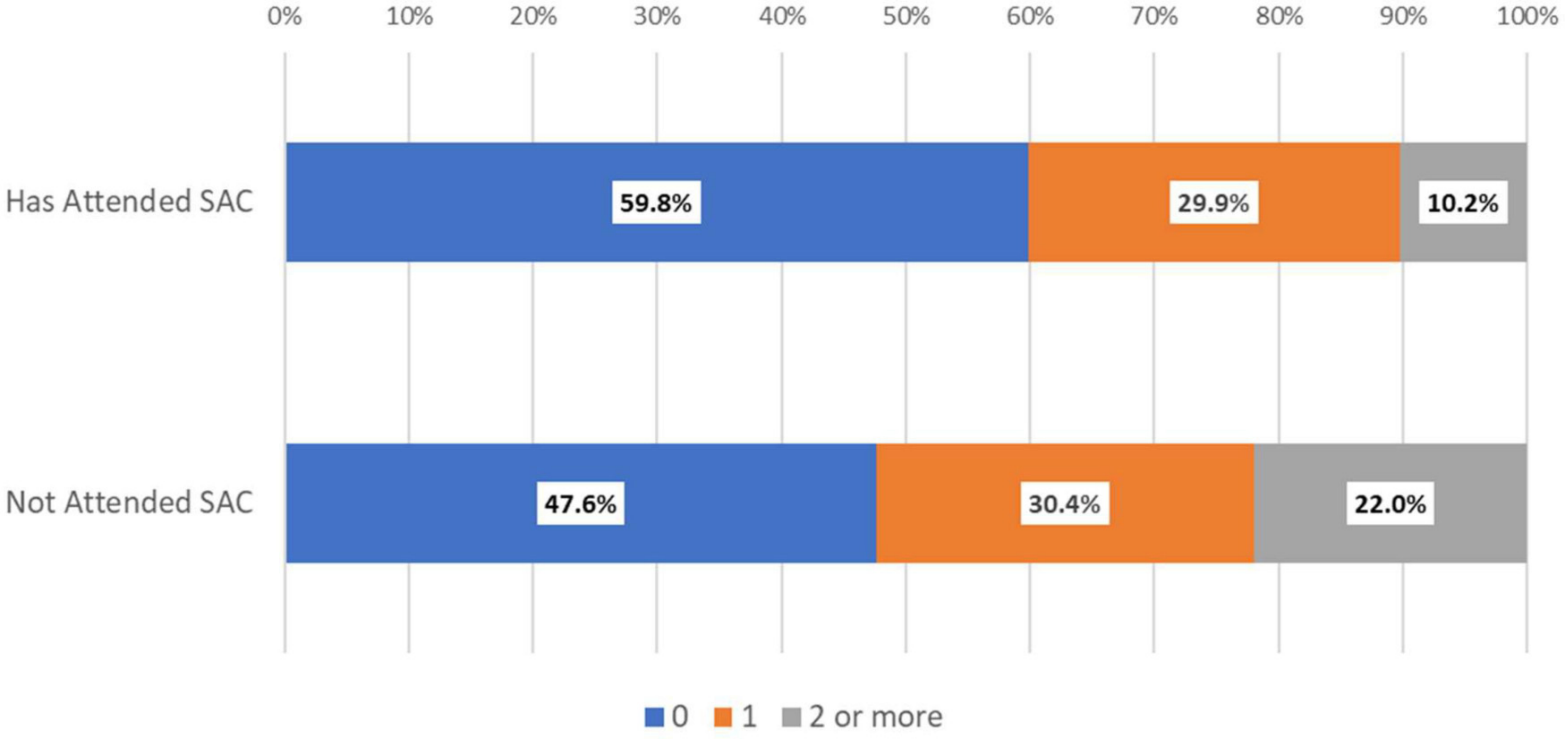
Attendance and count of NoN, ataxia related comorbidities (*N* = 432) SAC attendance was strongly associated with a lower count of comorbidities [Pearson chi-squared test *χ*^2^ (2, *N* = 432) = 12.44, *p* = .002].

Across the total cohort Chi-squared tests identified several statistically significant (<.01) associations. SAC attendance was strongly associated with a lower count of comorbidities. Only 10.2% of ‘Has Attended SAC’ reported two or more comorbidities in comparison to 22.0% in ‘Not Attended SAC’. In contrast, 59.8% of ‘Has Attended SAC’ reported no comorbidities vs. 47.6% for ‘Not Attended SAC’ (Figure 2).

### Travel time to SAC

**Figure 3:**
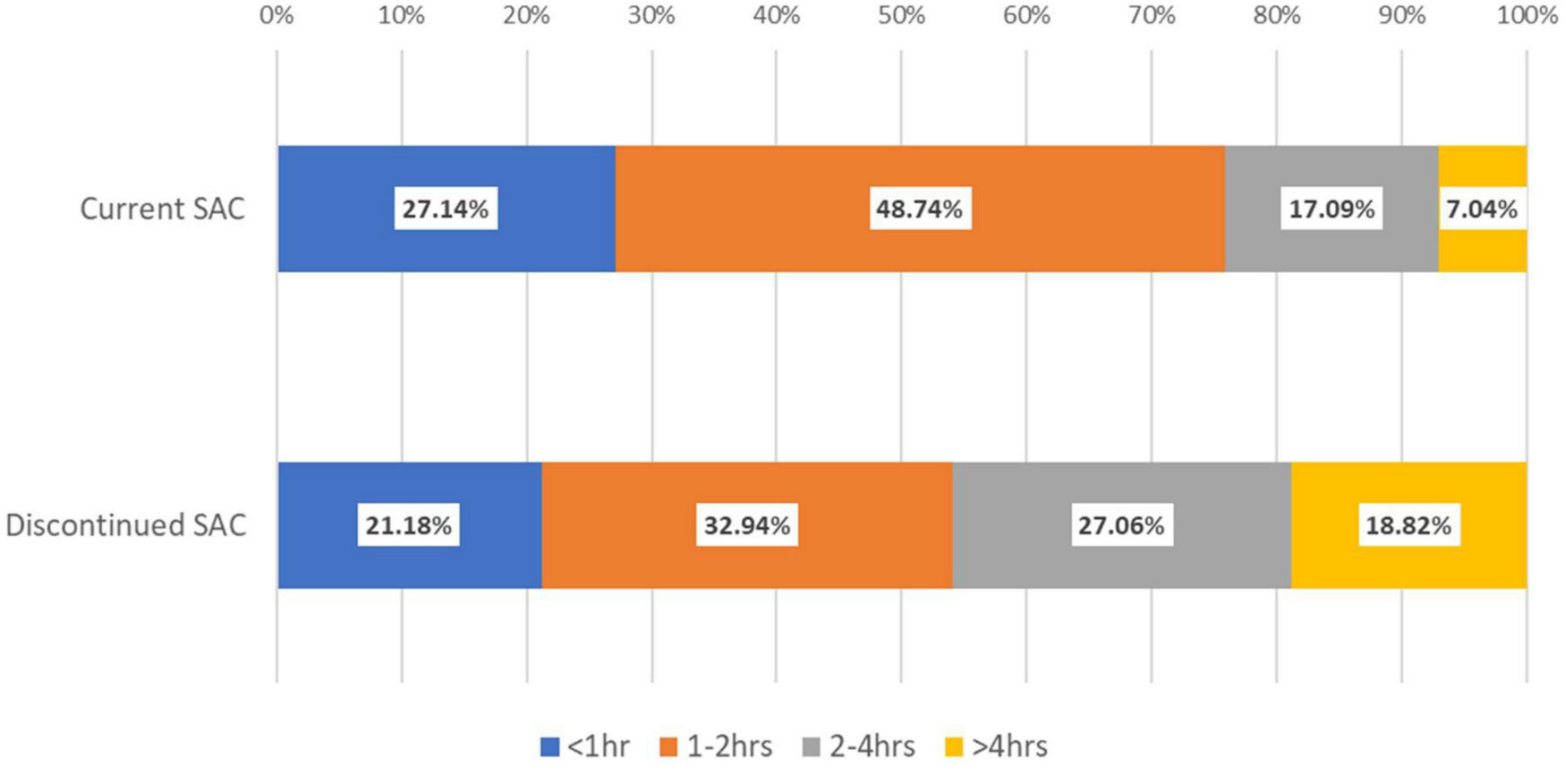
Travel time to SAC: current and discontinued SAC (*N* = 284) Increased travel time was associated with a reduction in the proportion of people attending SAC [Pearson chi-squared test *χ*^2^ (3, *N* = 284) = 15.00, *p* = .002].

There was a strong association between travel time and ‘Current SAC’ vs. ‘Discontinued SAC’ in the total cohort. Chi-squared tests identified a significant (<.01) association between increasing travel time and a reduction in the proportion of ‘Current SAC’ relative to ‘Discontinued SAC’ (Figure 3).

### Employment status

**Figure 4:**
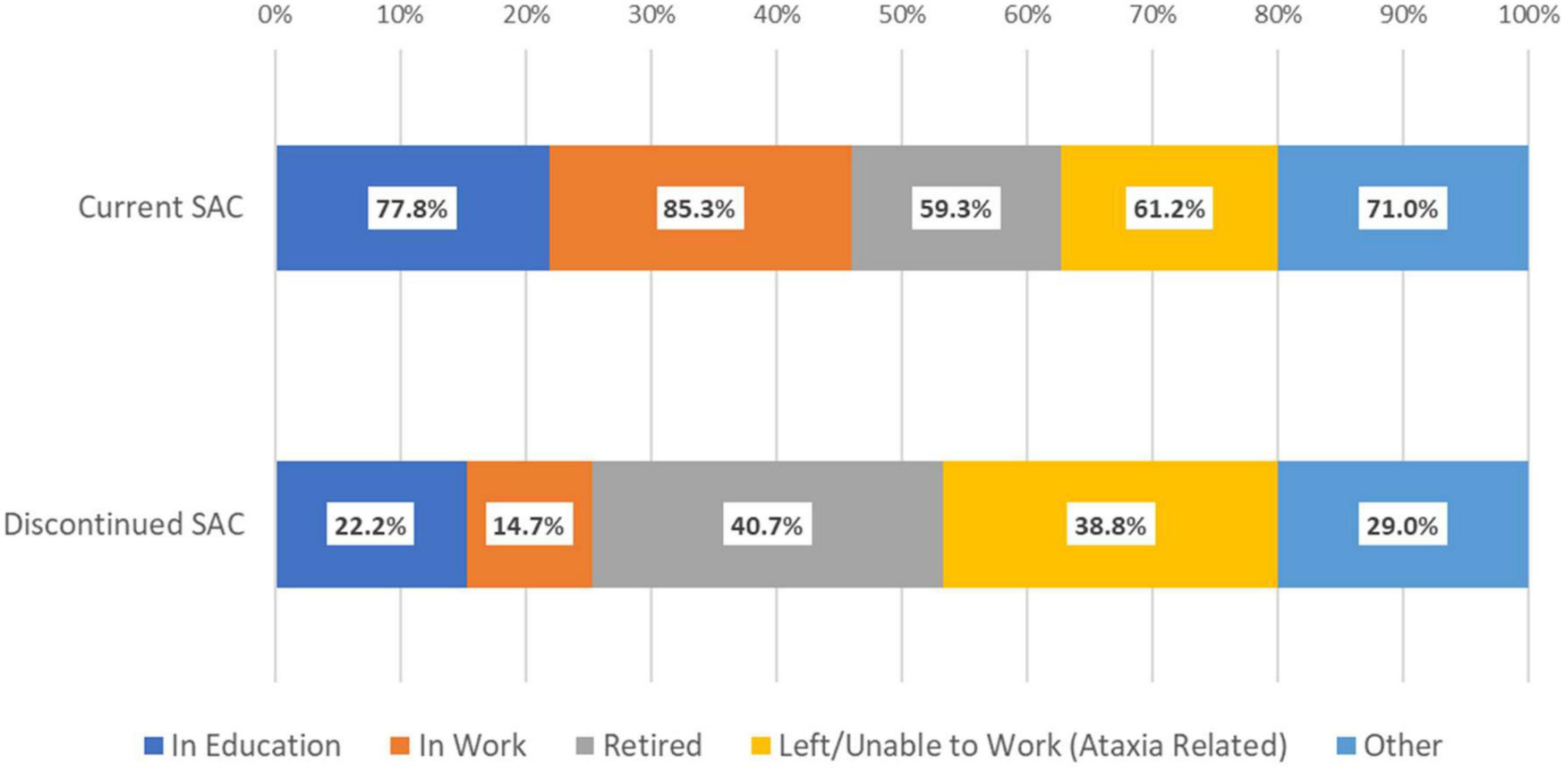
Employment status: current and discontinued SAC (*N* = 277) A significant association was found with a higher proportion of respondents in ‘current SAC’ in employment compared with ‘discontinued SAC’ respondents [Pearson chi-squared test *χ*^2^ (4, *N* = 277) = 15.26, *p* = .004]. The % labels sum to 100% for each colour category. For example, of respondents who were in education, 77.8% were categorised as “Current SAC” and the remaining 22.2% were “Discontinued SAC”.

There was a strong association between employment status and ‘Current SAC’ vs. ‘Discontinued SAC’ attendance in the total cohort. Chi-squared tests identified a statistically significant association (<.01) with a higher proportion of ‘Current SAC’ in employment than ‘Discontinued SAC’ (Figure 4).

### Mobility

**Figure 5:**
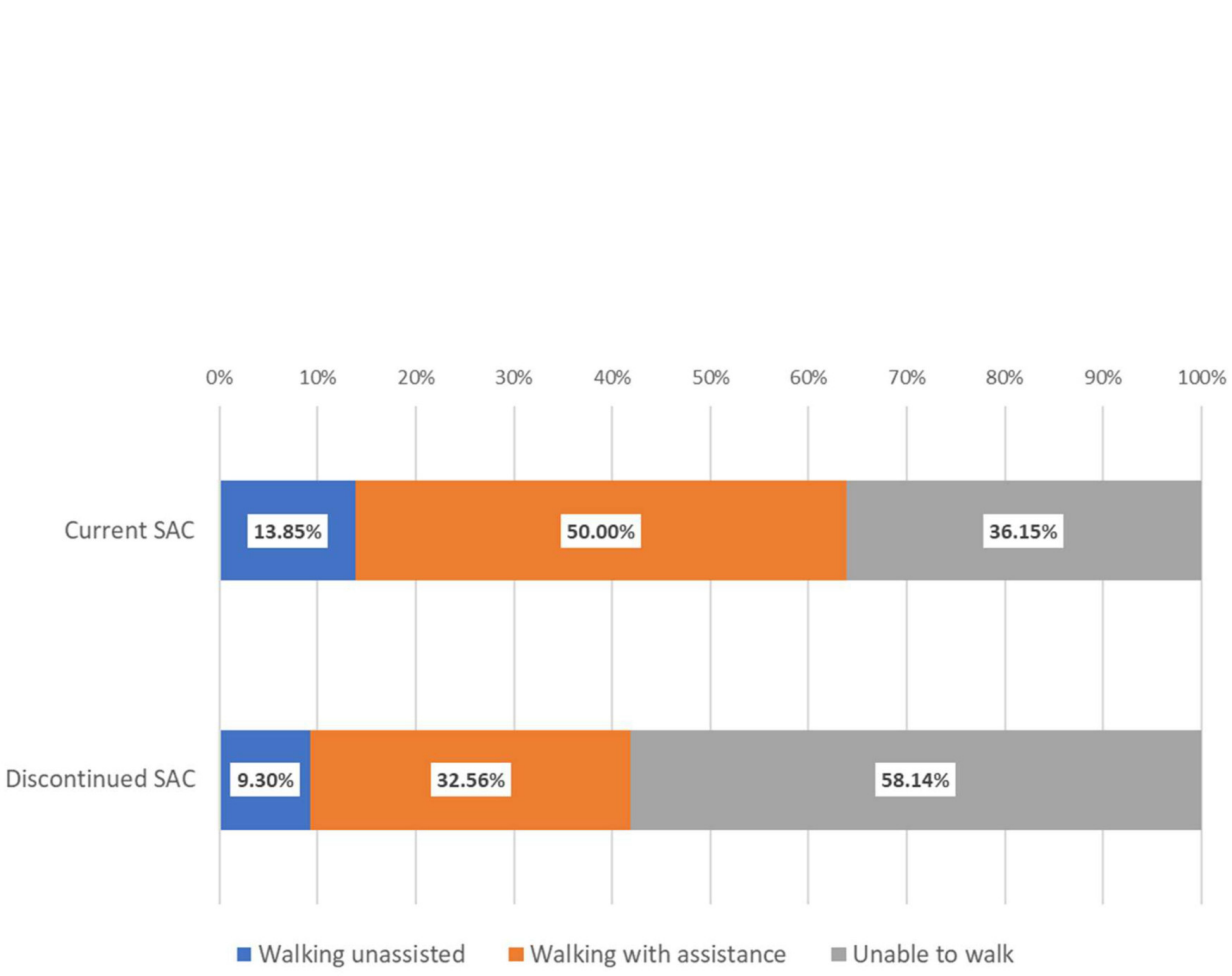
Mobility status: current and discontinued SAC (*N* = 173) A statistically significant association was found between ‘unable to walk’ and ‘discontinued SAC’ [Pearson chi-squared test *χ*^2^ (2, *N* = 173) = 6.43, *p* = .040].

The proportion in ‘Discontinued SAC’ was observed to increase relative to ‘Current SAC’ as mobility impairment increased. Chi-squared tests identified a statistically significant <.05 association between ‘Unable to Walk’ and ‘Discontinued SAC’ (Figure 5).

### Ataxia diagnosis

**Figure 6:**
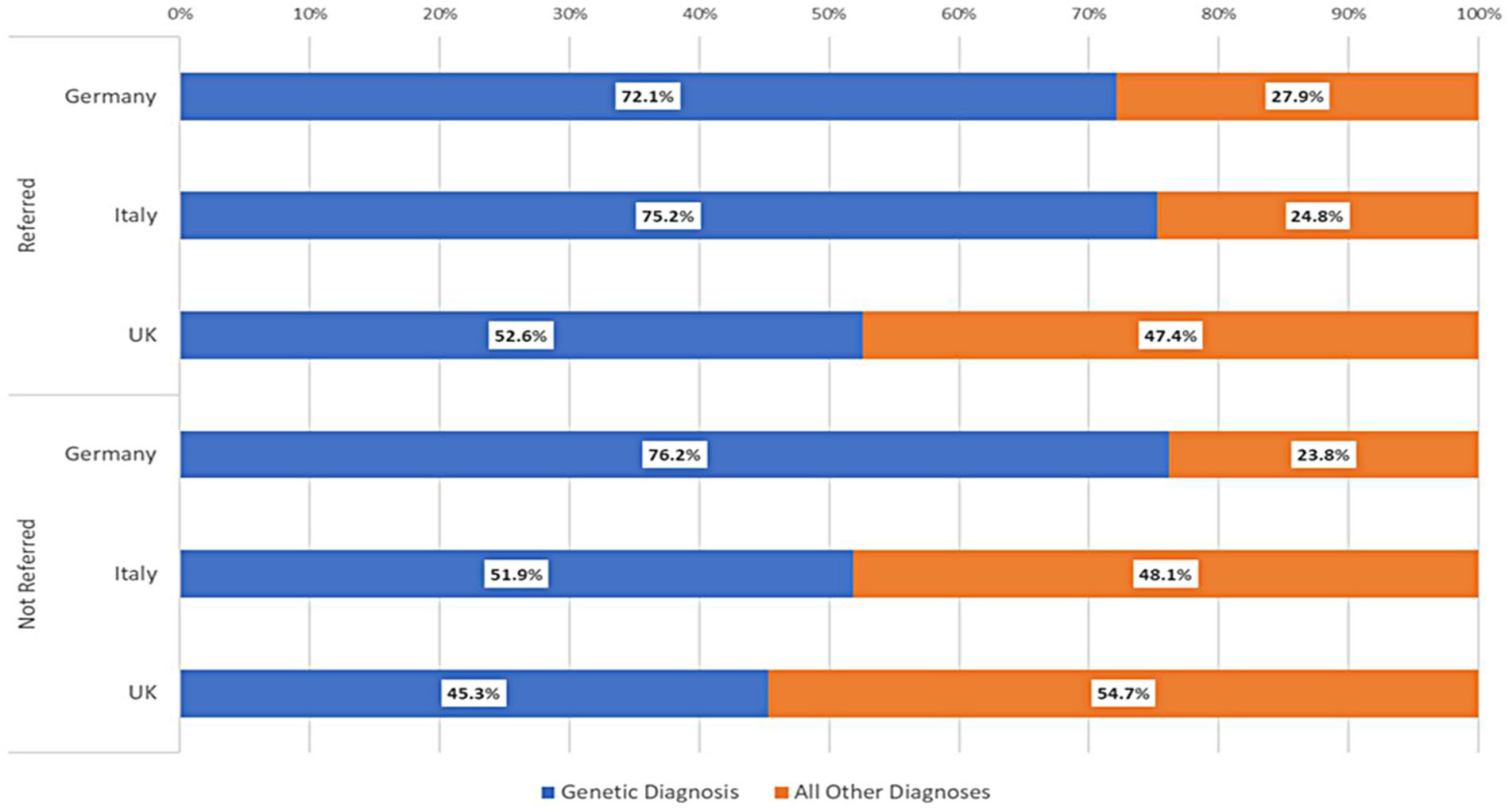
Genetic diagnosis and referral status by country (*N* = 481). A statistically significant association was found between respondents attending SAC and genetic diagnosis, with more respondents with genetic diagnosis in the referred category in Italy and Germany compared to the UK [Pearson chi-squared test *χ*^2^ (2, *N* = 481) = 25.16, *p* = <.001].

Statistically significant differences (<.01) were observed in referral to an SAC by ataxia diagnosis. Table 1 shows that much higher proportions of the survey respondents in Germany and Italy were referred to SACs than in the UK (71.8% and 80.9%, vs. 51.7%). This pattern was repeated when categorising whether a diagnosis indicated a hereditary ataxia (termed ‘Genetic Diagnosis’), or was a non-hereditary ataxia (‘All Other Diagnoses’). 72.1% of German respondents with a genetic diagnosis were in the ‘Referred’ category and in Italy it was 75.2%, whereas the UK was lower at 52.6% (Figure 6).

Analysis of the referral rates for each individual ataxia diagnosis at the country level also generated statistically significant associations. While the numbers were too small for EA to obtain a meaningful result, it was possible to use Fisher's Exact Test for the 4 more populous diagnoses. For FA the variation was most pronounced (*N* = 83, *p* = <.001, 2-sided), with all 14 FA cases in Germany being referred to an SAC and 40 out of 45 (88.9%) FA respondents in Italy being referred. Only half of the UK's 24 FA respondents indicated they had been referred to an SAC. The overall referral rate for FA was 79.5%. For Inherited CA (*N* = 194, *p* = .005, 2-sided) it was observed that Italy (42 of 51, 82.4%) referred a much higher proportion than the UK (55 of 99, 55.6%). Germany (28 out of 44, 63.6%) was in line with the overall referral rate for Inherited CA of 64.4%. For Idiopathic CA (*N* = 146, *p* = .036, 2-sided) it was observed that the UK (51 of 113, 51.3%) again had a significantly lower number of referrals relative to Germany and Italy (23 out of 33 combined, 69.7%). No significant association with referral status was observed for “Ataxia other types”.

When examining SAC attendance by ataxia diagnosis similar observations could be made. However, the division of respondents by SAC attendance status was slightly more even than by referral status. Respondents who have been referred to an SAC, but have yet to attend would be categorised in the ‘Not Attended SAC’ cohort, whereas they would qualify as ‘Referred’ under referral status. No significant association was observed between referral source or time since diagnosis and SAC attendance or referral, either for the total cohort or for any individual country.

## Discussion

This study of 550 participants with ataxia will expand the limited evidence base on SAC access in Germany, Italy and the United Kingdom. Potential factors associated with referral to an SAC or discontinued SAC access are identified and a number of disparities in access to SAC between the three countries are highlighted. These findings should be interpreted as exploratory, given the cross-sectional design and the characteristics of the surveyed populations. Interpretation of the results including cross-country comparisons requires careful consideration of potential selection bias due to methodological difference (see Limitations section).

Within these constraints, several associations were observed. The count of non-ataxia related comorbidities per respondent was observed to be significantly associated with SAC attendance, as those with fewer comorbidities were found to be more likely to attend an SAC. While the direction of this relationship cannot be determined from the present analysis, this finding is consistent with the literature for studies involving other disabling conditions, which have found poorer health to be an impediment to accessing health services (25, 26). This finding highlights the need for vigilance in monitoring for known disease-related comorbidities, such as Diabetes in FA, and reducing risk for secondary comorbidities such as cardiovascular disease to maximise ongoing SAC attendance (27). It could be hypothesised that SAC access may play a protective role in reducing comorbidity through health education and monitoring for known risk factors, however changes over time to investigate causality were beyond the scope of this cross-sectional study. It is unclear from the available data whether an increasing number of comorbidities hinders SAC access or conversely whether SAC attendance plays a role in limiting accumulation of comorbidities. These findings highlight the need for further longitudinal work to examine how comorbidity burden and service access interact over time.

Travel time to speciality services was also clearly associated with reduced access to an SAC. Cost and burden of travel are obvious barriers, a finding supported by some of our previous studies (16, 23) and by existing literature for other neurodegenerative conditions such as MS (28). Caution is required in interpretation however, as travel time may be linked to other unmeasured factors such as geography, service distribution, or individual circumstances. Factors hindering SAC attendance such as distance and travel can be mitigated. Further work in this area could include the measurement of distance and travel cost to identify locations where additional SACs would optimally improve access. Additionally, telehealth consultations, which utilise telephone or video conferencing technologies, have been employed effectively during the COVID pandemic (29), and could potentially be better exploited by this patient group. Telehealth could be employed in cooperation with primary care teams in the latter disease stages, when increasing comorbidity and disability make physical attendance at an SAC more difficult.

It should be acknowledged that this technology has possible limitations for SAC use. Where a definitive ataxia diagnosis has not yet been achieved, skilled neurological examination and specialised investigations should remain an essential part of the diagnostic pathway. Not all in-person examination can be replicated remotely. Further, ataxia-related impairment of speech, vision, hearing and cognitive function, may also pose significant challenges to accessing telehealth. A 2022 study in MS Telehealth care established generally positive uptake, however older age, lower socio-economic status and disease-related impairment (particularly moderate to severe visual symptoms, a pertinent issue for many with ataxia) were associated with reduced access to telehealth (29).

Unemployment was found to be high within the survey samples, with a greater proportion of ‘Current SAC’ in employment than ‘Discontinued SAC’, and greater loss of employment due to ataxia observed in the Discontinued SAC group. This may potentially reflect the financial burden of travel to an SAC, however, income data were not examined via these surveys, and it is therefore not possible to determine whether financial and socio-demographic factors were conclusive for discontinued SAC access. Additionally, employment findings should be interpreted with caution due to the correlation between age and employment. Once again the broad age categories limited the ability to draw any comparisons with respect to age-related unemployment statistics for the wider populations in each country.

The relationship between employment status and better clinical and disease management is well recognised in other chronic conditions (30). The high rates of unemployment in this survey sample therefore highlight the importance of improving uptake of vocational rehabilitation services which are known to significantly improve employment outcomes in other long term neurological conditions. Services such as employment support and assistive technology services are known to be significant predictors of improved employment outcomes (31).

The majority of Italian and German respondents reported limited mobility, with 86.4% being either unable to walk or requiring some form of assistance. Unsurprisingly, increasing mobility impairment, as observed in Figure 5, was associated with discontinued SAC attendance. While this should be interpreted cautiously due to an assumed relationship between age and deteriorating mobility, this finding is consistently reported in the literature for other neurological conditions, with disease progression and decreasing mobility associated with reduced specialised care access or not receiving any physician input (32). Such physical barriers further emphasise the need to explore telehealth alternatives to maximise continued SAC access.

Even allowing for the known geographic concentration of some hereditary ataxias, a wider than expected variation in the distribution of diagnosis types being referred to SACs was observed. The UK sample demonstrated a much lower representation of people with genetic diagnoses, in particular FA, being referred to SACs than European counterparts in contrast to known prevalence rates. The observed FA prevalence for example in the UK is 1:54,000 vs. 1:80,000–1:90,909 in Italy and 1:53,000–1:149,000 in Germany, with further variations in geographic concentrations reported within countries (33). This finding should be interpreted with caution however, as the survey sample may not be an accurate representation of the true SAC population within each country, with several confounding factors. It was beyond the scope of the available data to establish whether referral to an SAC resulted in a genetic diagnosis, or whether a genetic diagnosis triggered referral to an SAC. Genetic diagnoses would be expected to predominate if referral to an SAC is triggered via recruitment for clinical trials.

Respondents with Idiopathic CA reported a significantly lower rate of referrals to UK SACs than respondents in Germany and Italy. This may however simply reflect the higher representation of idiopathic responses in the UK survey, which in turn may be linked to variations in the distribution method of surveys.

Should the sample distributions for ataxia type attending SACs in each country not accurately represent the wider ataxia populations then this will impact on numerous other observations. FA, for example, would be expected to be a younger cohort due to the nature of disease progression, with onset of disease typically occurring in childhood, thereby influencing the age distribution by country (5). The lack of representation in the 80 + cohort in Italy and Germany may reflect a focus on FA patients (who are typically a younger cohort given the disease progression) for research purposes, which may in turn explain the absence of idiopathic and older respondents in these countries, as opportunities to participate in research may be lacking.

Also unexpected was the lack of association between impact of ataxia on daily living and SAC referral or attendance. In other neurological conditions increasing impairment is associated with an increased likelihood for regular specialist input (32), however in these survey samples those experiencing minimal impact on daily living were as likely to be attending or referred to an SAC as those experiencing constant problems. This highlights opportunities for SACs to maximise input that can improve outcomes for patients through early education and advice for optimally managing their condition.

No conclusions could be drawn regarding SAC access and time since diagnosis, as survey questions did not establish whether a diagnosis was achieved before referral to an SAC or after, hence the role that SACs play in the diagnostic process is not clear from the available data.

Overall, these findings should be interpreted within the context of an exploratory, cross-sectional, survey-based analysis using unadjusted bivariate comparisons. While the results identify associations that may be relevant to SAC access, they do not establish causal relationships. Further prospective, standardised and multinational studies are needed to better characterise patient pathways, account for potential confounding factors, and inform the development of equitable models of specialist care.

### Strengths and limitations

This study is based on a multinational study population, and included a broad range of variables covering socio-demographic and ataxia-specific factors. Multisite studies for rare diseases such as ataxia provide an opportunity to maximise enrolment and wider application of the findings, and facilitate collaboration among a larger group of expert clinicians (34). Limitations include self-reported data subject to recall and reporting bias, non-probabilistic sampling, unknown non-participation, and differing recruitment strategies, introducing potential for selection bias and limiting generalisability. Survey instruments differed between countries and required harmonisation, with variations in wording, response options and timing of data collection, including during the COVID-19 pandemic, potentially affecting comparability. Analyses were limited to unadjusted bivariate comparisons, with no multivariable modelling due to heterogenous survey instruments, sparse data in some categories, and inconsistent confounders. Without knowing the age at which people discontinue SAC access it is not possible to assess to what degree any shift from ‘Current SAC’ to ‘Discontinued SAC’ may be influenced by age progression. The closure of a third SAC in the UK in September 2018 meant that not all respondents who discontinued SAC attendance in this cohort did so by choice. This represented however a small number of respondents (*N* = 12), not likely to significantly impact the analysis. Finally, the cross-sectional design precluded assessment of temporality or causality, and associations should therefore be considered exploratory. Despite these limitations, the study provides one of the largest multinational datasets currently available on access to specialist ataxia services and offers novel insights into factors that may influence engagement with specialist care.

## Conclusions

This study defined key characteristics of respondents accessing SACs in the UK, Italy and Germany, highlighted differences between SAC and non-SAC respondent cohorts and identified national variations in SAC access. Respondents with a genetic diagnosis were found to have greater representation in SACs in Italy and Germany than in the UK. Ascertaining whether a definitive diagnosis was obtained before or after SAC attendance would contribute to a more accurate picture of respondent motivation to attend. Fewer comorbid conditions and being in employment were strongly associated with SAC attendance. These findings highlight where further investigation is required to promote improved SAC access. Shorter travel time to SAC was also strongly associated with SAC attendance. Telehealth in conjunction with primary care teams has the potential to transform SAC accessibility, particularly for those with a greater disease burden, and future work should explore applicability with this particular disease group.

The results of this study provide some understanding of the underlying drivers and impediments to accessing SACs. The ultimate goal of such work is to ensure equal health opportunities are available for all affected by these challenging conditions.

## Data Availability

The data analyzed in this study is subject to the following licenses/restrictions: The data that support the findings of this study are available from the corresponding author upon reasonable request. Requests to access these datasets should be directed to Paola Giunti, p.giunti@ucl.ac.uk.

## References

[1] De Silva R Greenfield J Cook A Bonney H Vallortigara J Hunt B. Guidelines on the diagnosis and management of the progressive ataxias. Orphanet J Rare Dis. (2019) 14(1):51. Published 2019 February 20. 10.1186/s13023-019-1013-9 30786918 PMC6381619

[2] Parkinson MH Boesch S Nachbauer W Mariotti C Giunti P. Clinical features of friedreich’s ataxia: classical and atypical phenotypes. J Neurochem. (2013) 126(Suppl 1):103–17. 10.1111/jnc.12317 23859346

[3] Diallo A Jacobi H Cook A Labrum R Durr A Brice A. Survival in patients with spinocerebellar ataxia types 1, 2, 3, and 6 (EUROSCA): a longitudinal cohort study. Lancet Neurol. (2018) 17(4):327–34. 10.1016/S1474-4422(18)30042-5 29553382

[4] Klockgether T Mariotti C Paulson HL. Spinocerebellar ataxia. Nat Rev Dis Primers. (2019) 5(1):24. Published 2019 April 11. 10.1038/s41572-019-0074-3 30975995

[5] Synofzik M Németh AH. Recessive ataxias. Handb Clin Neurol. (2018) 155:73–89. 10.1016/B978-0-444-64189-2.00005-6 29891078

[6] Salman MS. Epidemiology of cerebellar diseases and therapeutic approaches. Cerebellum. (2018) 17(1):4–11. 10.1007/s12311-017-0885-2 28940047

[7] Wardle M Majounie E Muzaimi MB Williams NM Morris HR Robertson NP. The genetic aetiology of late-onset chronic progressive cerebellar ataxia. A population-based study. J Neurol. (2009) 256(3):343–8. 10.1007/s00415-009-0015-2 19259763

[8] Sailer A Houlden H. Recent advances in the genetics of cerebellar ataxias. Curr Neurol Neurosci Rep. (2012) 12(3):227–36. 10.1007/s11910-012-0267-6 22527681

[9] Muzaimi MB Thomas J Palmer-Smith S Rosser L Harper PS Wiles CM. Population based study of late onset cerebellar ataxia in south east Wales. J Neurol Neurosurg Psychiatry. (2004) 75(8):1129–34. 10.1136/jnnp.2003.014662 15258214 PMC1739172

[10] Zesiewicz TA Wilmot G Kuo SH Perlman S Greenstein PE Ying SH. Comprehensive systematic review summary: treatment of cerebellar motor dysfunction and ataxia: report of the guideline development, dissemination, and implementation subcommittee of the American academy of neurology. Neurology. (2018) 90(10):464–71. 10.1212/WNL.0000000000005055 29440566 PMC5863491

[11] De Silva RN Vallortigara J Greenfield J Hunt B Giunti P Hadjivassiliou M. Diagnosis and management of progressive ataxia in adults. Pract Neurol. (2019) 19(3):196–207. 10.1136/practneurol-2018-002096 31048364 PMC6585307

[12] Lynch DR Schadt K Kichula E McCormack S Lin KY. Friedreich ataxia: multidisciplinary clinical care. J Multidiscip Healthc. (2021) 14:1645–58. Published 2021 Jun 28. 10.2147/JMDH.S292945 34234452 PMC8253929

[13] Rare Diseases Europe (EURORDIS). About rare diseases. Available from: http://www.eurordis.org/about-rare-diseases (Accessed March 13, 2023).

[14] Dharssi S Wong-Rieger D Harold M Terry S. Review of 11 national policies for rare diseases in the context of key patient needs. Orphanet J Rare Dis. (2017) 12(1):63. Published 2017 March 31. 10.1186/s13023-017-0618-0 28359278 PMC5374691

[15] Giunti P Morris S Relja M Pastores G Quoidbach V. Toward earlier diagnosis and treatment of rare neurological disorders: the value of coordinated care and specialist centers. Croat Med J. (2019) 60(2):156–7. 10.3325/cmj.2019.60.156 31044588 PMC6509631

[16] Vallortigara J Greenfield J Hunt B Hoffman D Reinhard C Graessner H. Patient pathways for rare diseases in Europe: ataxia as an example. Orphanet J Rare Dis. (2023) 18(1):328. 10.1186/s13023-023-02907-y 37848998 PMC10583310

[17] Debouverie M Laforest L Van Ganse E Guillemin F, LORSEP Group. Earlier disability of the patients followed in multiple sclerosis centers compared to outpatients. Mult Scler. 2009;15(2):251–7. 10.1177/1352458508097919 19181774

[18] Mansfield C Thomas N Gebben D Lucas M Hauber AB. Preferences for multiple sclerosis treatments: using a discrete-choice experiment to examine differences across subgroups of US patients. Int J MS Care. (2017) 19(4):172–83. 10.7224/1537-2073.2016-039 28835741 PMC5564278

[19] Saposnik G Camacho A Díaz-Abós P Brañas-Pampillón M Sánchez-Menéndez V Cabello-Moruno R. Therapeutic decision-making under uncertainty in the management of spinal muscular atrophy: results from DECISIONS-SMA study. Neurol Ther. (2022) 11(3):1209–19. 10.1007/s40120-022-00366-4 35657490 PMC9338192

[20] Chiu C Bishop M Pionke JJ Strauser D Santens RL. Barriers to the accessibility and continuity of health-care services in people with multiple sclerosis: a literature review. Int J MS Care. (2017) 19(6):313–21. 10.7224/1537-2073.2016-016 29270089 PMC5734715

[21] Minden SL Hoaglin DC Hadden L Frankel D Robbins T Perloff J. Access to and utilization of neurologists by people with multiple sclerosis. Neurology. (2008) 70(13 Pt 2):1141–9. 10.1212/01.wnl.0000306411.46934.ef 18362274

[22] European Brain Council. The Value of Treatment Policy White Paper: Towards optimizing research and care for brain disorders. Brussels: European Brain Council (2017). Available from: http://www.braincouncil.eu/wpcontent/uploads/2017/06/EBC_white_policy_paper_DEF26072017_Low.pdf (Accessed March 13, 2023).

[23] Morris S Vallortigara J Greenfield J Hunt B Hoffman D Reinhard C. Impact of specialist ataxia centres on health service resource utilisation and costs across Europe: cross-sectional survey. Orphanet J Rare Dis. (2023) 18(1):382. 10.1186/s13023-023-02971-4 38062507 PMC10704806

[24] Perez-Lloret S van de Warrenburg B Rossi M Rodríguez-Blázquez C Zesiewicz T Saute JAM. Assessment of ataxia rating scales and cerebellar functional tests: critique and recommendations. Mov Disord. (2021) 36(2):283–97. 10.1002/mds.28313 33022077

[25] Gell NM Mroz TM Patel KV. Rehabilitation services use and patient-reported outcomes among older adults in the United States. Arch Phys Med Rehabil. (2017) 98(11):2221–2227.e3. 10.1016/j.apmr.2017.02.027 28385481

[26] Elrod CS DeJong G. Determinants of utilization of physical rehabilitation services for persons with chronic and disabling conditions: an exploratory study. Arch Phys Med Rehabil. (2008) 89(1):114–20. 10.1016/j.apmr.2007.08.122 18164340

[27] Cnop M Igoillo-Esteve M Rai M Begu A Serroukh Y Depondt C. Central role and mechanisms of *β*-cell dysfunction and death in friedreich ataxia-associated diabetes. Ann Neurol. (2012) 72(6):971–82. 10.1002/ana.23698 23280845 PMC4900175

[28] Lechner-Scott J Agland S Giovannoni G Hawkes C Levy M Yeh EA. Inequality in accessing healthcare for people with MS. Mult Scleral Relat Disord. (2023) 72:104655. 10.1016/j.msard.2023.104655 36990053

[29] Marrie RA Kosowan L Cutter G Fox R Salter A. Disparities in telehealth care in multiple sclerosis. Neurol Clin Pract. (2022) 12(3):223–33. 10.1212/CPJ.0000000000001167 35747551 PMC9208413

[30] Vijayasingham L Mairami FF. Employment of patients with multiple sclerosis: the influence of psychosocial-structural coping and context. Degener Neurol Neuromuscul Dis. (2018) 8:15–24. Published 2018 March 26. 10.2147/DNND.S131729 30050385 PMC6053901

[31] Chiu CY Chan F Bishop M da Silva Cardoso E O'Neill J. State vocational rehabilitation services and employment in multiple sclerosis. Mult Scler. (2013) 19(12):1655–64. 10.1177/1352458513482372 23519974

[32] Barin L Kaufmann M Salmen A Kamm CP Gobbi C Kuhle J. Patterns of care for multiple sclerosis in a setting of universal care access: a cross-sectional study. Mult Scler Relat Disord. (2019) 28:17–25. 10.1016/j.msard.2018.11.033 30530118

[33] Vankan P. Prevalence gradients of friedreich’s ataxia and R1b haplotype in Europe co-localize, suggesting a common palaeolithic origin in the franco-Cantabrian ice age refuge. J Neurochem. (2013) 126(Suppl 1):11–20. 10.1111/jnc.12215 23859338

[34] Fiss AL McCoy SW Bartlett DJ Chiarello LA Palisano RJ Stoskopf B. Sharing of lessons learned from multisite research. Pediatr Phys Ther. (2010) 22(4):408–416. 10.1097/PEP.0b013e3181faeb11 21068641

