# Supplementary figures and images for: Access to specialist ataxia centres in Europe: cross-sectional surveys in the United Kingdom, Italy, and Germany
Source: Front Health Serv. 2026 Jun 24;6:1787866. doi: 10.3389/frhs.2026.1787866 (PMC13341664; doi:10.3389/frhs.2026.1787866)

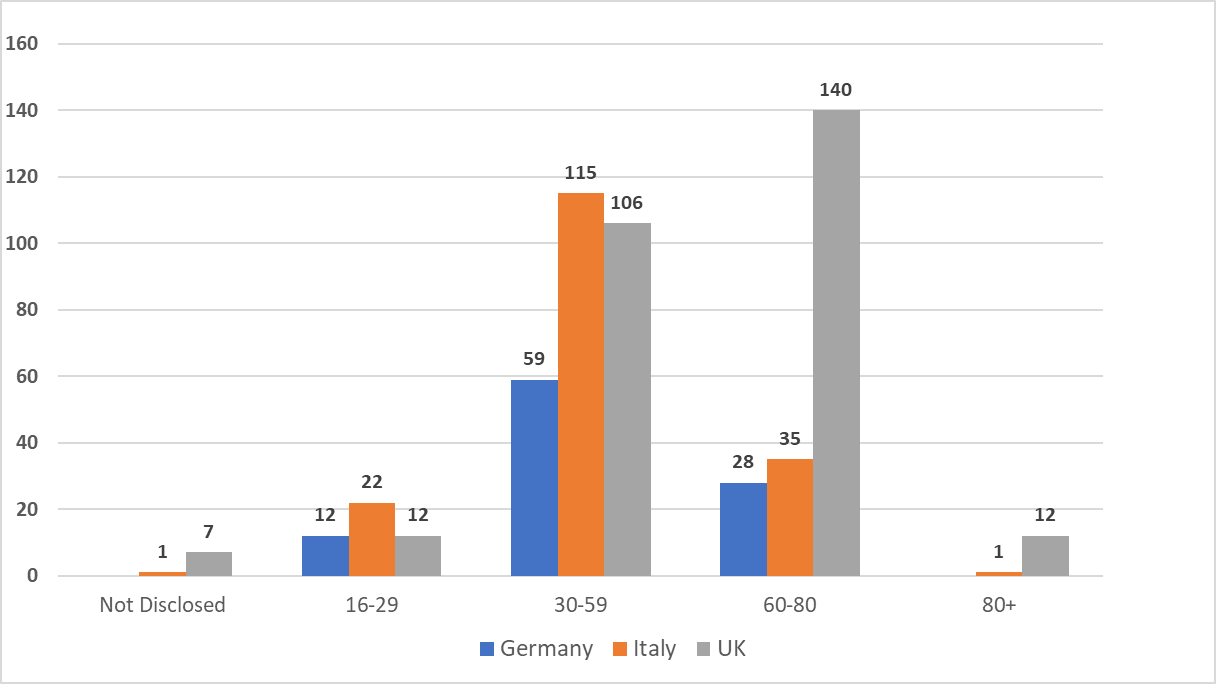

Supplement: Supplementary file 4 [file Image1.png]

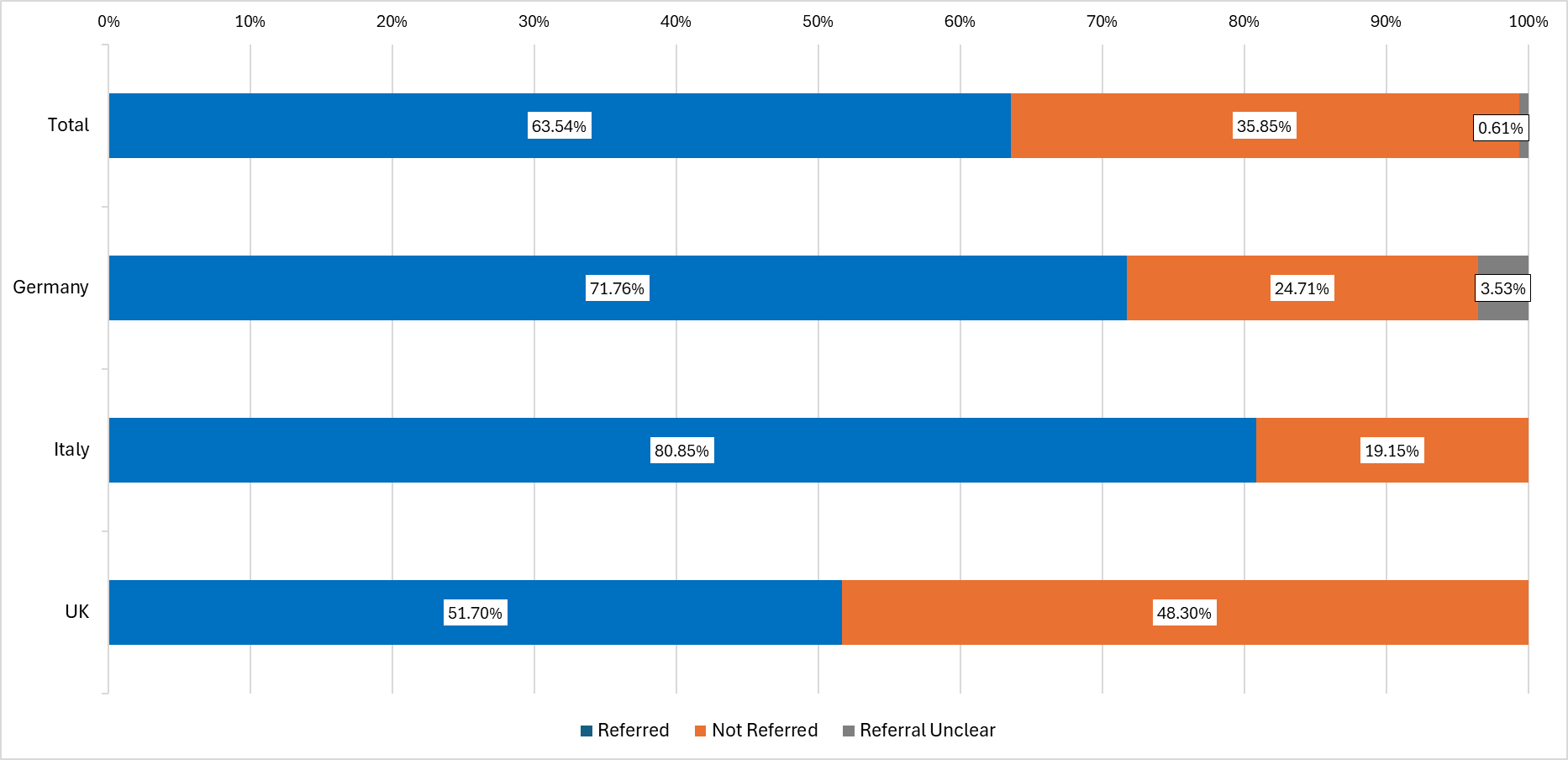

Supplement: Supplementary file 5 [file Image2.png]
